# Collaboration with World Health Organization

**DOI:** 10.4102/phcfm.v11i1.2175

**Published:** 2019-09-19

**Authors:** Shabir Moosa

**Affiliations:** 1 Department of Family Medicine and Primary Care, Faculty of Health Sciences, University of the Witwatersrand, Johannesburg, South Africa; 2 WONCA Africa, Johannesburg, South Africa

You have had a chance to get to know the World Organisation of Family Doctors in Africa, WONCA Africa in a previous editorial and to see WONCA Africa plans and achievements since October 2018 (published elsewhere in this journal). There is a particularly important collaboration that has been growing and that family doctors in Africa need to understand: the WONCA collaboration with World Health Organization (WHO).

World Organisation of Family Doctors has been in official relations as a non-state actor with WHO for many years, with liaison between WHO (currently via Dr Shannon Barkely) and WONCA (currently via Prof. Viviana Martinez-Bianchi), many exchanges and communications, and a slow but growing relevance for family doctors. The Astana Declaration was an important event in October 2018, just after Dr Donald Li became President of WONCA in the WONCA World Conference in Seoul, South Korea. World Organisation of Family Doctors World leaders participated in the many deliberations around it, including the development of the declaration before it. There are many important commitments to primary healthcare (PHC) including that PHC is a cornerstone of a sustainable health system for universal health coverage (UHC), that promotive, preventive, curative rehabilitative and palliative care must be accessible to all, that PHC must meet all people’s health needs across the life course with services that are continuous, integrated, people-centred and with empowering multisectoral action. Whilst signatories commit to increased funding of PHC and a PHC workforce, with an appropriate skill mix, there was reluctance to mention family doctors as part of the PHC team in the Astana Declaration, despite the many deliberations mentioning the value of family doctors in the PHC team, especially in the context of Africa (refer to Astana Declaration).

Dr Donald Li, as President of WONCA, strove to address this with Dr Tedros Adhanom Gebyreseus (Director-General of WHO) in subsequent months. The result was a Memorandum of Understanding (MOU) that was signed by them on 21 January 2019 at the time of the WHO Executive Board Meeting in Geneva (https://www.woncaafrica.org/post/wonca-mou-with-who-1). It commits both organisations to collaborate on realising universal health coverage and PHC, especially the central role of family doctors in the delivery of primary care. There is a strong commitment to a broad range of family medicine training. This will be operationalised with further exchange of letters (a less formal process) based on agreements at national, regional and global levels. There will be an annual report summarising activities in support of this MOU.

Dr Donald Li and the WONCA team ensured that relations were developed between WONCA and WHO in Africa (WHO AFRO) since October 2018 at Astana and then with WHO Executive Board and World Health Assembly meetings. We reached out to Dr Prosper Tumusiime of WHO AFRO in December 2018 and had a very engaging discussion. Dr Tumusiime committed to join us at the Kampala Conference and we explored a visit to Brazzaville to meet the WHO AFRO team. Unfortunately, the visit was not possible but Dr Prosper Tumusiime joining us from beginning to end of our conference has been a major milestone for WONCA Africa.

We had a workshop on WHO-WONCA Collaboration in Africa at the conference and Dr Tumusiime viewed the MOU as a major step forward for relations between WHO AFRO and WONCA Africa. We developed a good understanding of WHO AFRO. The WHO AFRO has a regional office in Brazaville and three sub-regional inter-country teams in Harare, Ougadougou and Libreville. The Regional Director, Dr Matshidiso Moeti, has a team of eight in her executive in Brazzaville: Dr Joseph Cabore (Programme Management) coordinating four technical clusters/programmes (Dr Tumusiime – Health Systems Strengthening, Dr Felicitas Zawaira – Family and Reproductive Health, Dr Steven Shongwe – Non-communicable Diseases and Dr Magaran Bagoyoko – Communicable Diseases). There is also Dr Kisolo Chisaka (Director for the Office of the Regional Director), Mr Raul Thomas (General Management and Coordination) and Dr Ibrahim Soce Fall (Health Security and Emergencies). Dr Tumusiime (heading the Health Systems Strengthening Cluster) seems most appropriate as the WONCA liaison. He has a number of departments under him: health information, knowledge management and research; health technology; health policies and strategy; service partnerships and delivery systems (including human resources, district health services, etc.). He explained that WHO country offices (WCOs) were spread across the continent and are open to approach (https://www.afro.who.int/about-us).

We agreed that there would be a deliberate introduction of WCOs to WONCA member organisations (MOs) by country and a request for their facilitation of a high-level meeting between MOs and government ministries around the role of family doctors in that country. We believe that this will spur more countries to form MOs. I have been invited to join and observe the WHO Regional Committee for Africa 19–23 August 2019. All 47 ministers of health of the region participate. He has kindly allowed the President-elect Dr Dan Abubakar to join me. Dr Tumusiime is also planning for a team of 6–10 from WONCA Africa to meet with various departments in Brazzaville in the few days before this. We have also been requested to comment on a document he is crafting around an African framework for district health services and to be presented to the Regional Committee Meeting.

We also had many explicit commitments by conference workshop participants to help WHO AFRO with guideline development, international advocacy, consulting, capacity-building/training and research. There were many more suggestions on how we could work together, including developing an African Forum for Primary Care involving all members of the PHC team. We wish to take all these suggestions and ideas up in further deliberations with WHO AFRO and translate them in the next few months into workplans for which formal letters can be exchanged.

Dr Tumusiime was present as we crafted the Kampala Commitment 2019: a commitment by family doctors to universal health coverage, PHC and building the capacity of PHC teams at scale in Africa (https://www.woncaafrica.org/post/kampala-commitment-2019). We will use this to advocate for a clear role for family doctors in African PHC, especially for the United Nations High-Level Meeting on universal health coverage on 23 September 2019 (https://www.who.int/news-room/events/detail/2019/09/23/default-calendar/un-high-level-meeting-on-universal-health-coverage). He is very aware of the commitment, youth and dynamism that exist amongst family doctors in Africa, and our global friends, and we look forward to build on these relations.

I would like to share some recent key WHO AFRO documents and our clearly shared vision for PHC in Africa in the next editorial. Check the WONCA Africa website at www.woncaafrica.org, and @WONCAAfrica on Facebook, Twitter and Telegram to keep more immediately abreast of happenings in WONCA Africa.

